# GABA_B_ Encephalitis: A Fifty-Two-Year-Old Man with Seizures, Dysautonomia, and Acute Heart Failure

**DOI:** 10.1155/2015/812035

**Published:** 2015-11-02

**Authors:** Matthew C. Loftspring, Eric Landsness, Lindsey Wooliscroft, Robert Rudock, Sally Jo, Kevin R. Patel

**Affiliations:** Department of Neurology, Washington University, St. Louis, MO 63110, USA

## Abstract

Autoantibodies to the *γ*-aminobutyric acid receptor, subtype B (GABA_B_), are a known cause of limbic encephalitis. The spectrum of clinical manifestations attributable to this antibody is not well defined at the present time. Here we present a case of GABA_B_ encephalitis presenting with encephalopathy, status epilepticus, dysautonomia, and acute heart failure. To our knowledge, heart failure and dysautonomia have not yet been reported with this syndrome.

## 1. Introduction

GABA_B_ encephalitis refers to limbic encephalitis caused by autoantibodies to the *γ*-aminobutyric acid receptor, subtype B (GABA_B_). Its clinical presentation is similar to other limbic encephalitides and Morvan syndrome, with psychiatric symptoms and seizures predominating. However, the range of clinical manifestations attributable to this antibody has not yet been fully described owing primarily to its recent discovery and its rarity. Here, we present a case of GABA_B_ encephalitis with additional components of acute heart failure and dysautonomia.

## 2. Case Presentation

A previously healthy 52-year-old Caucasian man was admitted to our hospital with a subacute, progressive syndrome of refractory seizures, psychosis, dysautonomia, and encephalopathy. He initially presented to an outside facility with new-onset seizures, but after multiple hospitalizations, and despite two antiseizure medications, the patient continued to have breakthrough seizures. Two weeks later, he gradually developed amnesia, cognitive difficulties, visual hallucinations, paranoia, and anxiety, requiring a readmission to evaluate and treat for a presumed primary psychiatric condition. In spite of one month of antiepileptic drug adjustments he continued to have breakthrough seizures, prompting transfer to our institution. On exam he was somnolent with poor attention. He was oriented to self, location, and year but was unable to perform basic arithmetic; the remainder of his neurologic exam was nonfocal.

**Figure 1:**
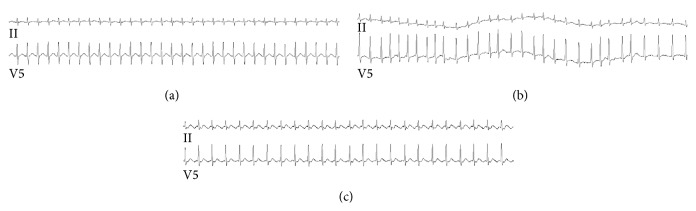
Representative electrocardiograms demonstrating tachyarrhythmias observed in this case. (a) Supraventricular tachycardia with a ventricular rate of 195 beats per minute. (b) Atrial fibrillation with rapid ventricular response. (c) Atrial flutter with a 2 : 1 conduction block.

An infectious etiology was investigated, which included blood, urine, tracheal aspirate, and CSF cultures, but was negative. His vital signs were persistently abnormal during the first ten days after his transfer: temperature up to 38.3°C, respiratory rate up to 32 breaths per minute, and sustained heart rates up to 122 beats per minute. The patient's hospital course was further complicated by heart failure and hypotension, necessitating critical care monitoring and an epinephrine infusion. On presentation to the intensive care unit his troponin I was 0.26 ng/mL which downtrended to 0.15 ng/mL and was undetectable within 24 hours (the lower limit of detection on our assay is 0.03 ng/mL). Electrocardiograms revealed a supraventricular tachycardia; there were intermittent episodes of atrial flutter with 2 : 1 atrioventricular nodal conduction block and atrial fibrillation with rapid ventricular response (Figure 1). Furthermore, a transthoracic echocardiogram demonstrated severe mitral regurgitation, depressed left ventricular function, and an ejection fraction of 26%. Amiodarone and metoprolol were consequently started with return to normal sinus rhythm.

**Figure 2:**
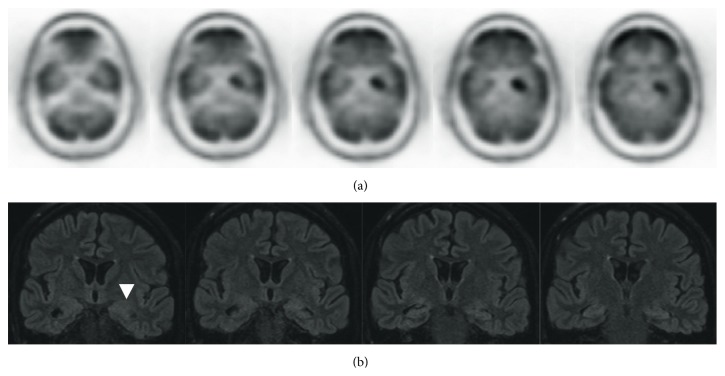
(a) Axial PET demonstrating significant uptake of FDG in the left mesial temporal lobe. (b) Coronal FLAIR MRI with subtle hyperintensity surrounding the left hippocampus (arrowhead).

A brain MRI showed FLAIR hyperintensities in left hippocampal body with surrounding mild edema (Figure 2). Continuous video electroencephalogram monitoring captured frequent electrographic and clinical seizures of left posterior temporal onset and moderate to severe generalized slowing. A lumbar puncture was performed with an opening pressure of 20 cmH_2_O; cerebrospinal fluid studies showed 4 nucleated cells/*μ*L, protein of 35 mg/dL, glucose of 85 mg/dL, and zero oligoclonal bands. Paraneoplastic panel in the CSF was positive for anti-GABA_B_ receptor antibody. This antibody panel is performed at the Mayo Clinic Laboratories (Rochester, MN, USA) and utilizes indirect immunofluorescence on animal brain slices to screen for antibodies reactive to brain antigens. Positive results are further characterized and reflex tests for other autoreactive antibodies are performed based on the staining pattern. Reflex autoantibody tests include those against the NMDA receptor, AMPA receptor, and GAD-65 which were not detected; therefore direct testing for these autoantibodies did not occur. Other relevant antibodies with this presentation are anti-LGI1 anti-GABA_A_; however these were not screened or tested. Negative antibodies on this panel were ANNA-1, ANNA-2, ANNA-3, anti-glial nuclear antibody, anti-Purkinje cell cytoplasmic antibody, types 1 and 2 and Tr, anti-amphiphysin, and anti-CRMP-5.

An autoimmune workup was negative for ENA and ANCA, but with a mildly positive ANA (1 : 160). Anti-thyroid peroxidase and thyroglobulin antibodies were elevated at 2910 units/mL and 4.8 ng/mL, respectively. These latter two antibodies are increasingly being appreciated as nonspecific markers of autoimmune processes in what is often called “steroid responsive encephalopathy.” Thyroid stimulating hormone was elevated at 5.78 mIU/L, but free T4 was normal at 1.53 *μ*g/dL. Whole-body CT and PET scan showed no evidence of malignancy but did reveal markedly increased FDG uptake within the medial left temporal lobe (Figure 2).

The patient was initially treated with high-dose IV methylprednisolone at 1 gram per day for six days, in addition to plasma exchange. Shortly after treatment there was decreased seizure frequency and continued maintenance of normal sinus rhythm. He was given a dose of rituximab and started on a twelve-week prednisone taper. His encephalopathy and psychosis were slower to resolve, requiring intermittent symptomatic treatment. At the time of discharge he had no electrographic evidence of epileptic activity on a regimen of lacosamide, levetiracetam, carbamazepine, and scheduled lorazepam. A repeat transthoracic echocardiogram demonstrated resolution of systolic heart failure with a normal ejection fraction of 67%. One month after discharge, a repeat brain MRI revealed a decrease in the left temporal FLAIR signal; two months after discharge, MRI revealed complete resolution.

## 3. Discussion

GABA_B_ encephalitis occurs relatively infrequently; however the characterization of the antibody and clinical phenotype has occurred recently. Interestingly, it associated with small cell lung cancer in 50–80% of patients from recent case series [1]. This is the most common neoplasm described with this syndrome and implies that surveillance should continue for several years after the onset of the encephalitis. By contrast, dysautonomia occurs more frequently in limbic encephalitis such as with anti-NMDA autoantibodies [2]. Höftberger et al. have previously reported autonomic dysfunction in one of twenty patients with GABA_B_ syndrome [3]. We are not aware of acute heart failure in the context of GABA_B_ encephalitis, though there is at least one report of Takotsubo cardiomyopathy in a patient with limbic encephalitis [4]. This was presumed to be a paraneoplastic process associated with B cell lymphoma but unfortunately an autoantibody was not identified. Cardiogenic shock and heart failure have also been seen in rhombencephalitis caused by enterovirus 71 [5]. In that report there was no PCR evidence of enterovirus 71 in seven hearts examined for pathology. Similarly there was no significant cardiac inflammatory infiltrate, all suggesting that heart failure was due to a neurogenic mechanism rather than myocarditis. The cardiac dysfunction seen in these cases globally affected the left ventricle, similar to the patient described in the present report.

Although the known etiologies of supraventricular tachycardia and acute heart failure are numerous and varied, the clinical circumstances in this case strongly suggest that they were a consequence of neurologic injury. Three points when taken together support this: (1) GABA_B_ receptors are transcribed in the fetal but not adult heart and are thought to be found primarily in the nervous system, though they have recently been described in smooth muscle of the human aorta [6, 7]; (2) the cardiomyopathy persisted until the patient underwent immunosuppressive treatment and then fully resolved with treatment; (3) there are few etiologies of his cardiomyopathy identified which would be expected to resolve with immunosuppression or spontaneously, though autoimmune myocarditis or tachycardia-induced cardiomyopathy is among them.

In our patient there was hypermetabolism in the left medial temporal lobe, determined by FDG PET imaging which is suggestive of inflammation. This is consistent with limbic encephalitides that have been reported in the literature. While common, it is not a uniform finding. Indeed, hypermetabolism has been reported in the bilateral medial temporal lobes, occipital lobes, frontal lobes, and cerebellum. There are also cases of hypometabolism though this seems to occur in older patients and may be associated with concurrent neurodegenerative processes [8]. The specific brain structures that are inflamed are expected to relate to the clinical manifestations of the limbic encephalitis. For example, our patient had prominent difficulty with cognitive function, seizures, and hallucinations, all of which could be attributed to the temporal lobe or via its connections. Similar presentations of GABA_B_ encephalitis have been described in a recent large case series [3].

The connections between the medial temporal lobe and the insular cortex provide one pathway by which limbic encephalitis can lead to dysautonomia [4, 9]. The insular cortex is known to modulate autonomic pathways from the brain to the heart. For example, cardiac complications such as arrhythmias, myocardial infarction, and heart failure are reported after left insular stroke as well as intracerebral and subarachnoid hemorrhages [10, 11]. Additionally, other stroke locations such as hemispheric and basal ganglia as well as epilepsy have also been associated with heart failure, suggesting that there are many connections involved in neural regulation of the heart [12, 13]. Further support of a neural-mediated cardiac injury in this patient is promoted by animal studies where baclofen injection into the tract of the nucleus solitarius produced hypertension and tachycardia and inhibited the depressor baroreflex response [14]. Others have similarly found that vagal inhibition by the solitary tract is mediated by both GABA_A_ and GABA_B_ receptors [15].

One mechanism of heart failure and dysautonomia in this case may be due to blockade GABA_B_ receptors in the brainstem, ultimately leading to failure of vagal inhibition of cardiac function. Another possibility is that this was tachycardia-associated cardiomyopathy, which in turn could be due to dysautonomia caused by the limbic encephalitis. Alternative explanations for our patient's heart failure include direct antibody mediated effects on the myocardium. However, there are no reports of GABA receptors in the myocardium. Also, anti-thyroid peroxidase antibodies are not associated with heart failure [16, 17]. Nonetheless, we can only speculate about the precise pathogenesis of heart failure. A cardiac MRI or myocardial biopsy would have been helpful in identifying an autoimmune myocarditis, due to either anti-GABA_B_ antibodies biding to sites other than the GABA_B_ receptor or additional antibodies that we did not detect. Fortunately, the patient's cardiac function was improving with treatment and we did not feel that further diagnostic testing of the heart would change management; therefore it was not pursued.

Takotsubo cardiomyopathy, the most widely recognized to be associated with neurologic injury, is thought to be mediated by increased levels of circulating catecholamines and in turn increases in intracellular calcium and in systemic vascular resistance and afterload [12, 18]. Takotsubo cardiomyopathy is primarily characterized by apical ballooning, a feature that was absent in this case [19]. As such, this entity is unlikely in our patient, who instead had global systolic dysfunction.

In this report we highlight two uncommon complications of GABA_B_ encephalitis: acute heart failure and dysautonomia. To our knowledge, cardiomyopathy has not previously been recognized as a manifestation of this condition and dysautonomia is uncommon [20]. GABA_B_ encephalitis is associated with a varied clinical phenotype. We believe that this report further expands the clinical manifestations of this relatively uncommon syndrome.
